# Reemergence of pertussis in the highly vaccinated population of the Netherlands: observations on surveillance data.

**DOI:** 10.3201/eid0604.000404

**Published:** 2000

**Authors:** H E de Melker, J F Schellekens, S E Neppelenbroek, F R Mooi, H C Rümke, M A Conyn-van Spaendonck

**Affiliations:** Department of Infectious Disease Epidemiology, National Institute of Public Health and the Environment, Bilthoven, the Netherlands. H.de.melker@rivm.nl

## Abstract

We analyzed pertussis reporting, death, hospitalization, and serodiagnostic data from 1976 to 1998 to help explain the cause of the 1996 pertussis outbreak in the Netherlands. The unexpected outbreak was detected by an increase in pertussis reporting and by other surveillance methods. In 1996, according to reporting and serologic data, the increase in pertussis incidence among (mostly unvaccinated) children less than 1 year of age was similar to the increase in hospital admissions. Among older (mostly vaccinated) persons, the increase in hospital admissions was relatively small. The increase in pertussis incidence was higher among vaccinated than among unvaccinated persons of all ages. This resulted in lower estimates of vaccine effectiveness. The proportion of pertussis infections resulting in recognizable symptoms may have increased among vaccinated persons because of a mismatch of the vaccine strain and circulating Bordetella pertussis strains. The small immunogenicity profile of the Dutch vaccine may have resulted in greater vulnerability to antigenic changes in B. pertussis.


### Reemergence of Pertussis in the Highly Vaccinated Population of the Netherlands: Observations on Surveillance Data

**Hester E. de Melker, J.F.P. Schellekens, S.E. Neppelenbroek, F.R. Mooi, H.C. Rümke, and M.A.E. Conyn-van Spaendonck**
National Institute of Public Health and the Environment,
Bilthoven, the Netherlands

We analyzed pertussis reporting, death, hospitalization, and serodiagnostic data from 1976 to 1998 to help explain the cause of the 1996 pertussis outbreak in the Netherlands. The unexpected outbreak was detected by an increase in pertussis reporting and by other surveillance methods. In 1996, according to reporting and serologic data, the increase in pertussis incidence among (mostly unvaccinated) children less than 1 year of age was similar to the increase in hospital admissions. Among older (mostly vaccinated) persons, the increase in hospital admissions was relatively small. The increase in pertussis incidence was higher among vaccinated than among unvaccinated persons of all ages. This resulted in lower estimates of vaccine effectiveness. The proportion of pertussis infections resulting in recognizable symptoms may have increased among vaccinated persons because of a mismatch of the vaccine strain and circulating *Bordetella pertussis* strains. The small immunogenicity profile of the Dutch vaccine may have resulted in greater vulnerability to antigenic changes in *B. pertussis*.

The incidence of pertussis has been greatly reduced by mass vaccination; however, even in countries with high vaccination coverage, the disease is reemerging (1-4). A sudden increase in cases reflecting a pertussis outbreak in the Netherlands in 1996 (5) could not be explained by a decrease in vaccination coverage, which remained stable at 96% for at least three vaccinations in the first year of life. Until January 1999, children were vaccinated at 3, 4, 5, and 11 months of age with a diphtheria, tetanus, pertussis, and inactivated polio vaccine. In 1999, the schedule changed, and vaccine was administered at 2, 3, 4, and 11 months of age. The vaccine used meets international standards; no sign of an abrupt or gradual deterioration of vaccine quality, as determined at product release by the mouse protection test, was found. The introduction of vaccination against *Haemophilus influenzae* type b in 1993 did not interfere with the immunoresponse to pertussis (6), and no cohort effect in children vaccinated for *H. influenzae* type b was observed (5). A mismatch between the vaccine and circulating strains of *Bordetella pertussis* (5,7-9) may have contributed to pertussis reemergence.

Determining the epidemiology of pertussis by case-reporting data is hampered by changes in case definitions, availability and interpretation of laboratory diagnostic tests, case-reporting rates, and diagnostic practice. To ascertain the current epidemiology of pertussis in the Netherlands and to try to determine the cause of the 1996 epidemic, we compared case-reporting data from January 1976 to September 1998 with other surveillance data (deaths, hospitalizations, and positive serodiagnoses).

Address for correspondence: H.E. de Melker, Department of Infectious Diseases Epidemiology, National Institute of Public Health and the Environment, P.O. Box 1, 3720 BA Bilthoven, the Netherlands; fax: 31-30-274-4409; e-mail: H.de.melker@rivm.nl.

### Methods

### Surveillance Data

#### Case Reporting

Data from pertussis reporting (required by law since 1976) were obtained from 1976 to 1998 from the Inspectorate of Health. A case definition, introduced in 1988, included clinical symptoms and laboratory confirmation (or close contact with a person with laboratory-confirmed pertussis). The clinical symptoms are a serious cough >2 weeks, coughing attacks, or coughing followed by vomiting and at least one of the following: apnea, cyanosis, characteristic cough with whooping, subconjunctival bleeding, or leukocytosis. From 1988 to April 1997, laboratory confirmation was defined as either a positive culture of *B. pertussis* (or *B. parapertussis)* or positive two-point serology, in turn defined as a significant rise of immunoglobulin (Ig) G antibodies against pertussis toxin or IgA antibodies against *B. pertussis* in paired sera. In April 1997, a positive polymerase chain reaction (PCR) and positive one-point serology were also accepted as laboratory confirmation. Positive one-point serology was defined as high IgG or IgA antibody titers in a single serum sample.

From 1976 to 1988, only aggregated data were available on the total number of patients per year. These data included case-reporting date, number of vaccinated (at least three vaccinations) and unvaccinated or incompletely vaccinated patients, and number of patients with unknown vaccination status, with age at the time of report, by age-group (<1, 1-4, 5-9, and ≥10 years). From 1989 to 1992, the age (in years) and date of reporting were available for individual patients, while the aggregated data on vaccination status were similar to those of 1976 to 1988. Since 1993, the date of onset of symptoms, date of birth, age in years at the time of reporting, vaccination status, method of laboratory diagnosis, and contact with a person with laboratory-confirmed pertussis were included in the database. The method of laboratory diagnosis was differentiated as "microbiologic" (positive culture or PCR), "serologic," "epidemiologic" (i.e., contact with a patient with laboratory-confirmed pertussis), and "unknown."

The case distribution from 1976 to 1988 could only be assessed by date of reporting. From 1989 to 1992, the date of onset of symptoms was estimated by finding the median duration between date of first symptoms and date of reporting (1993 to 1994). This median duration (81 days) was then subtracted from the reporting date of the case at hand. The distribution of cases in 1989 to 1992 was based on this estimate, and age distribution was based on age at the time of reporting. For 1993 to September 1998, the distribution of cases was based on the date of first symptoms available in the database, and the age distribution was based on age at onset of symptoms.

#### Hospitalizations and Deaths

The number of hospitalizations with pertussis as the main diagnosis (ICD-9-CM 033) in 1976 to 1997 was obtained from the registry of the Foundation Information Center for Health Care. For 1989 to 1997, data were available by age group (<1, 1-4, 5-9, 10-14, 15-19, ≥20 years). The number of deaths (by age, in 5-year increments) caused by pertussis in 1976 to 1997 was obtained from the Central Bureau of Statistics.

#### Serology

Data on pertussis serology were obtained from the National Institute of Public Health and the Environment, the only laboratory in the Netherlands that performed serologic tests for suspected-pertussis patients from 1982 through January l, 1998. Other laboratories have performed an estimated 10% to 15% of serologic tests since 1998.

Serologic testing consisted of measuring IgA antibodies against a crude cell-wall preparation of *B. pertussis* (available since 1981) and IgG antibodies against purified pertussis toxin (available since 1984) in enzyme-linked immunosorbent assays, according to described methods that have not changed (10,11). The potency of the used reference sera was stable. Serologic interpretations have varied over the years. In 1982 to 1988, mostly single serum specimens were submitted. Since vaccination with the Dutch whole-cell vaccine only induces low levels of IgG against pertussis toxin (IgG-PT) and no IgA against *B. pertussis* (IgA-Bp), detection of IgA-Bp (sonicated mixture of the two strains included in the Dutch whole-cell vaccine) or moderate, high, or very high IgG-PT was reported as supportive of pertussis.

By 1987, it became clear that low and moderate IgG-PT and IgA-Bp levels were present in a large proportion of the population and that the prevalence increased with age. Therefore, in 1988, serologic interpretation of single serum specimens was abandoned, and only significant increase of IgG-PT or IgA-Bp in paired sera was considered confirmation of pertussis. The same year, a strict case definition for pertussis reporting was introduced, which included "positive two-point serology" in the laboratory-confirmation criteria. However, in 1993, when serum specimens from the population, vaccinees, and pertussis patients were tested, high IgG-PT or high IgA-Bp levels (greater than an age-specific cut-off value) were found to be very rare in the population (<2.5%). Such levels were not induced by vaccination, were present in at least 90% of patients with PCR- or culture-confirmed pertussis, and decreased again within 6 months to levels below the cut-off value (12-14). Since 1994, the laboratory-reported detection of such high values in one or both samples of a serum pair indicates "possible pertussis," although the case definition for reporting remained unchanged. From April 1997, the detection of such high levels in patients' samples was formally defined as "positive one-point serology" and was included in the case definition for reporting as laboratory-confirmed pertussis.

From the serologic database, we retrieved data on patients whose date of disease onset was January 1989 to September 1998 and for patients whose date of serologic result was January 1986 to December 1987. Patients with positive two-point serology in 1989 to 1998 and patients with positive one-point serology in 1994 to 1998 were selected. The criteria we recently defined for positive one-point serology were retrospectively applied to the serologic data of 1986–1987 and 1989–1993. The distribution of cases in 1986 and 1987 was calculated based on the year of the test result and, in 1989–1998, based on the year of first symptoms.

### Data Analysis

We used Epi-Info version 6.04 to estimate vaccine effectiveness[1] in persons ages 1 to 4 and 5 to 9 years (reporting data from 1976 to 1997), assuming an average vaccine coverage in the Dutch population of 96%. We compared completely vaccinated persons (at least three vaccinations) with incompletely vaccinated or unvaccinated persons (15).

Since positive serologic results are included in the case definition for reporting, the serodiagnosis and reported-case databases are not independent sources. For 1993 to 1997, the reported-case database and the database with records of serodiagnosis were linked at the individual-patient level to verify the type of serodiagnosis (positive two-point or positive one-point serology) on which the reporting was based. The completeness of the reported-case database was calculated from the proportions of reported patients with positive two-point and one-point serologic results. We used the statistical package SAS to analyze the data.

### Results

### Reported Cases, Hospitalizations, and Deaths, 1976 to September 1998

In the first years of mandatory reporting of pertussis cases, the case count was the lowest (Table 1). From 1983 to 1987, after immunoassays for pertussis serology became available, the number of reported cases increased yearly. In 1988, the year in which a case definition was introduced and positive serology was restricted to an increase in titer in paired sera, the number of reported cases declined sharply. Somewhat greater numbers of cases were reported in 1989-90 and 1993-94. In 1996, the number of cases was 12 times higher than in 1995, while a twofold decrease from the 1996 number was observed in 1997.

The number of reported cases from January to September 1998 (n = 1,582) was lower than that of the same periods in 1996 (n = 2,171) and 1997 (n = 2,004) but approximately six times higher than the average number of reported cases in the same months of 1989 to 1995 (n = 269).

The trend of hospitalizations was similar to that of case reports; however, the ratios varied by period (Table 1). This ratio was below one from 1976 to 1984, increased to 5.7 in 1987, and decreased sharply to 1.2 in 1988; it remained relatively stable from 1989 to 1995 but increased to 8.2 in 1996 and 6.1 in 1997.

From 1976 to 1997, seven deaths caused by pertussis were reported: one in 1981, two in 1993, two in 1996, and two in 1997. They occurred among children <1 year of age, except for one death in 1993, which occurred in the 5- to 9-year age group. According to the number of

[1]Vaccine effectiveness = 1 – (proportion of vaccinated cases/1 – proportion of vaccinated cases) X (1 – proportion vaccinated in the population/proportion vaccinated in the population).

Table 1. Reported cases, hospitalizations, ratios of reported cases to hospitalizations, positive 2-point serology and positive 1-point serology, 1976–1998

| Yr | Reports | Hosps | Pos. 2-point test | Pos. 1-point test | Ratio of reports/ hosps | Ratio of reports/pos. 2-point test | Ratio of reports/pos. 1-point test |
|---|---|---|---|---|---|---|---|
| 1976[a] | 4 | 52 | NA | NA | 0.1 | NA | NA |
| 1977 | 25 | 93 | NA | NA | 0.3 | NA | NA |
| 1978 | 1 | 32 | NA | NA | 0.0 | NA | NA |
| 1979 | 26 | 43 | NA | NA | 0.6 | NA | NA |
| 1980 | 30 | 65 | NA | NA | 0.5 | NA | NA |
| 1981[b] | 50 | 76 | NA | NA | 0.7 | NA | NA |
| 1982 | 80 | 98 | NA | NA | 0.8 | NA | NA |
| 1983 | 200 | 223 | NA | NA | 0.9 | NA | NA |
| 1984[c] | 534 | 200 | NA | NA | 2.7 | NA | NA |
| 1985 | 1,522 | 313 | NA | NA | 4.9 | NA | NA |
| 1986 | 2,159 | 388 | NA | 2,717 | 5.6 | NA | 0.8 |
| 1987 | 2,709 | 474 | NA | 3,295 | 5.7 | NA | 0.8 |
| 1988[d] | 112 | 92 | NA | NA | 1.2 | NA | NA |
| 1989 | 523 | 221 | 350 | 1,760 | 2.4 | 1.5 | 0.3 |
| 1990 | 397 | 157 | 278 | 1,204 | 2.5 | 1.4 | 0.3 |
| 1991 | 145 | 82 | 110 | 411 | 1.8 | 1.3 | 0.4 |
| 1992 | 160 | 101 | 238 | 861 | 1.6 | 0.7 | 0.2 |
| 1993 | 346 | 288 | 482 | 1,489 | 1.2 | 0.7 | 0.2 |
| 1994 | 519 | 276 | 498 | 1,867 | 1.9 | 1.0 | 0.3 |
| 1995 | 341 | 162 | 272 | 1,070 | 2.1 | 1.3 | 0.3 |
| 1996 | 4,231 | 513 | 1,885 | 7,854 | 8.2 | 2.2 | 0.5 |
| 1997[e] | 2,671 | 436 | 924 | 4,107 | 6.1 | 2.9 | 0.7 |
| 1998[f] | 1,582 | NA | 294 | 2,187 | NA | 5.4 | 0.7 |

[a]Mandatory case reporting introduced.
[b]IgA assay introduced.
[c]IgG assay introduced.
[d]Case definition for reporting (including strict criteria for interpretation of serologic tests) introduced.
[e]Formal acceptance of positive one-point serologic test.
[f]Data from January to September 1998.
1-point test = cases with one-point serology; 2-point test = cases with two-point serology; NA=not available.

hospitalizations among children <1 year of age, the case-fatality rates amounted to 0.1% on average in 1989 to 1995, 0.6% in 1996, and 0.7% in 1997.

### Serologic Results

The cases with positive one-point serology followed a trend similar to that of cases reported in 1986 to 1987 and 1989 to 1998 (Table 1). In 1986 and 1987, the ratio of reported cases to cases with positive one-point serology was highest (0.8); it decreased in 1989, and remained relatively stable from 1989 to 1996 (0.2 to 0.4). In 1996 to 1998, this proportion increased from 0.5 to 0.7.

The trends of cases with positive two-point serology and reported cases were similar (Table 1). From 1989 to 1995, the number of cases in each database was similar (ratio 0.7 to 1.5); in 1996 and 1997 (ratio 2.2 and 2.9) and particularly in 1998 (ratio 5.4), the number of reported cases was higher than the number of cases with positive two-point serology.

### Age-Specific Incidence, from Case Reporting and Hospitalizations, 1989–1997

In 1989 to 1995, the average annual incidence from case reporting was highest for infants <1 year of age. Such data for 1993 to 1995 show that the age-specific peak incidence occurred among ≤ 5-month-old infants. For infants <1 year of age, the incidence in 1996 was four times higher than the average incidence from 1989 to 1995 and 13 times higher than the incidence for older age groups (Figure 1). The age-specific peak incidence shifted to 4-year-old children in 1996 and 1997.

From 1993 to 1997, when the method of laboratory diagnosis was available for reported cases, similar shifts in age distribution were observed for cases confirmed by microbiologic

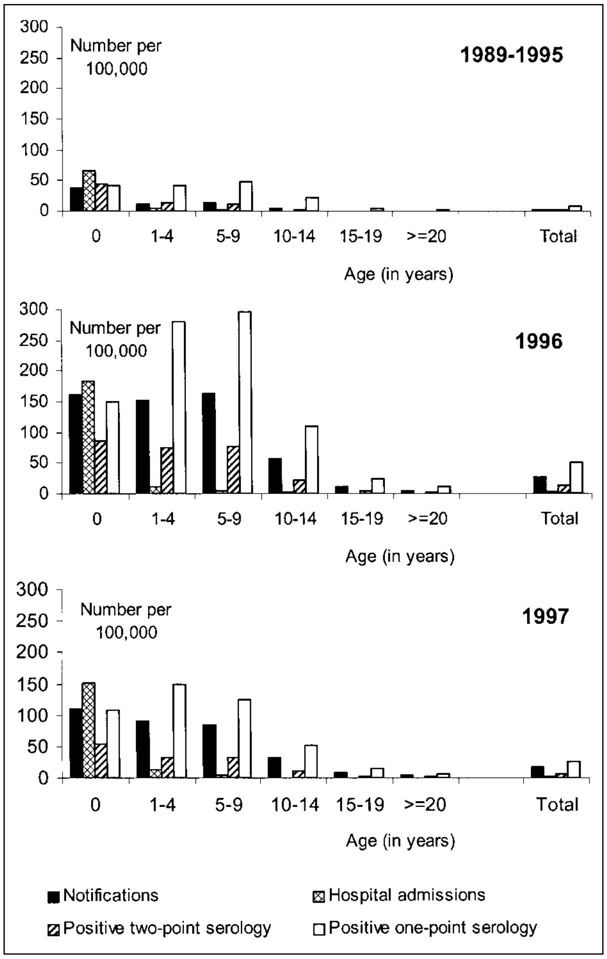

Figure 1. Average annual age-specific incidence (number per 100,000) in 1989 through 1995, in 1996, and in 1997.

method (i.e., culture or PCR), cases with positive two-point serology, and cases with positive one-point serology. However, reported cases confirmed microbiologically were those of the youngest patients; cases confirmed with one-point serology were those of the oldest patients. In contrast to reported cases, the greatest age-specific incidence of hospitalizations occurred among infants <1 year of age from 1989 to 1997 (Figure 1). The increase in incidence in 1996 was more stable for the various age groups.

### Age-Specific Incidence, from Serologic Results, 1989-1997

As in case reports, because of a relatively greater increase of pertussis among older age groups, the peak incidence among cases with positive two-point serology occurred among ≤5-month-old infants from 1989 to 1995 and among 4-year-old children in 1996 and 1997. The incidence of cases with positive one-point serology was greatest among 7-year-old children in 1989 and shifted towards 4-year-old children from 1992 to 1997. As with case reports, the increase for infants <1 year old was smaller (fourfold) than that for older age groups (fivefold to sevenfold) (Figure 1).

### Seasonal Trend

In March and April 1996, reported cases started to increase (Figure 2). The largest monthly number of cases occurred later in the year (October 1996) than in 1989-1995 and 1997-

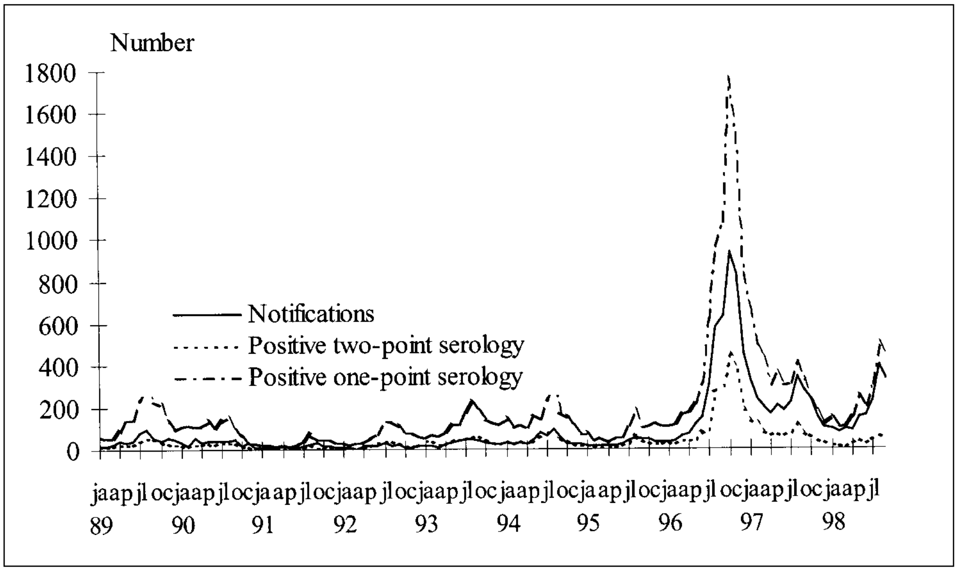

Figure 2. Reported pertussis cases, cases with positive two-point serology, and cases with positive one-point serology, per month, 1989–1998.

1998 (mostly in August, sometimes in July or September). The seasonal trend of positive two-point serology and positive one-point serology was similar to that of case reports (Figure 2).

### Vaccination Status of Reported Cases

According to case reports, vaccine effectiveness was high in 1981 to 1984 (1 to 4 years of age: 94% to 99%; 5 to 9 years of age: 87% to 100%) and in 1988 to 1993 (1 to 4 years of age: 89% to 95%; 5 to 9 years of age: 78% to 89%). The estimates were somewhat lower in 1985 to 1987 (72% to 85% for children 1 to 4 years of age and 56% to 77% for children 5 to 9 years of age). The estimates decreased after 1993 (Table 2), were lowest in 1996, and could not be determined in 1997 since the proportion of vaccinated patients exceeded 96%.

Table 2. Reported cases according to vaccination status and estimate of vaccine-effectiveness according to method of diagnosis

| Yr. | Method of diagnosis | 1- to 4-yr-olds (no.) | Vaccine efficacy (%)[a] | 5- to 9-yr-olds (no.) | Vaccine efficacy (%)[a] |
|---|---|---|---|---|---|
| 1993 | Microbiologic | 14 | 93 | 14 | 94 |
| | 2-point serology | 25 | 91 | 28 | 75 |
| | 1-point serology | 24 | 79 | 26 | 50 |
| | Other[b] | 35 | 96 | 20 | 90 |
| | Total | 98 | 93 | 88 | 84 |
| 1994 | Microbiologic | 23 | 56 | 15 | 42 |
| | 2-point serology | 36 | 67 | 36 | 83 |
| | 1-point serology | 63 | 71 | 65 | c |
| | Other[b] | 37 | 89 | 29 | 91 |
| | Total | 159 | 77 | 145 | 72 |
| 1995 | Microbiologic | 14 | 85 | 9 | 67 |
| | 2-point serology | 27 | 82 | 19 | 64 |
| | 1-point serology | 39 | 64 | 45 | c |
| | Other[b] | 37 | 53 | 27 | 67 |
| | Total | 117 | 72 | 100 | 45 |
| 1996 | Microbiologic | 116 | 67 | 123 | 3 |
| | 2-point serology | 245 | c | 288 | 49 |
| | 1-point serology | 545 | c | 782 | c |
| | Other[b] | 290 | 66 | 355 | 40 |
| | Total | 1,196 | 34 | 1,548 | 16 |
| 1997 | Microbiologic | 63 | 51 | 68 | 57 |
| | 2-point serology | 110 | c | 131 | c |
| | 1-point serology | 283 | c | 345 | c |
| | Other[b] | 244 | c | 260 | c |
| | Total | 700 | c | 804 | c |

[a]Estimated vaccine effectiveness; a vaccine coverage of 96% was used to estimate the incidence and vaccine effectiveness.
[b]Epidemiologic, serologic (differentiation between positive two-point serology and positive one-point serology not possible), clinical, or method of diagnosis unknown.
[c]Vaccine effectiveness could not be estimated as the percentage vaccinated was more than 96%.

Vaccine effectiveness for almost all methods of diagnosis was greater in 1993 than in 1994 to 1997 (Table 2). The decreasing trend was not consistent with all methods of diagnosis. Estimates for cases diagnosed microbiologically tended to be the highest; estimates for cases confirmed by one-point serology tended to be the lowest.

### Method of Diagnosis for Case Reports, 1993–1997

Linkage of the case-report and serodiagnosis databases for 1993 to 1997 showed that the proportion of reported cases confirmed microbiologically, epidemiologically, or by two-point serology, decreased, while the proportion of cases confirmed with positive one-point serology increased (Figure 3). The proportion of cases confirmed serologically but not matched with the serologic database also increased. Differentiating these cases by positive one-point and positive two-point serology was not possible. During 1996 and 1997, the proportion of cases confirmed microbiologically by quarter year was similar (8.7% to 10.2%), except for a smaller proportion (6.4%) in the last quarter of 1997. The proportion of cases confirmed by two-point serology was 12% to 19%. In the first two quarters of 1996 (34.9% to 35.6%) and in the fourth quarter of 1997 (34.3%), the proportion of cases confirmed by positive one-point serology was similar to the proportions for 1994 and 1995. The numbers increased to >50% in the fourth quarter of 1996 and the first quarter of 1997. The proportion of cases confirmed serologically that could not be matched with the serodiagnostic database was highest (35.3%) in the fourth quarter of 1997.

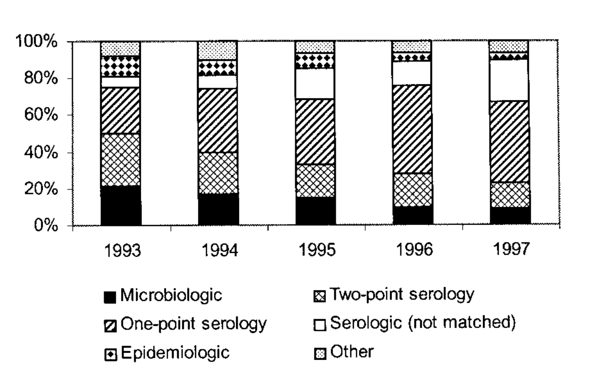

Figure 3. Method of diagnosis for reported pertussis cases from 1993 through 1997.

### Estimated Rate of Case Reporting

The reported proportion of cases with positive two-point serology increased from 24% in 1993 to 26% in 1994, 28% in 1995, and 42%-43% in 1996 and 1997. A similar trend was observed for the various age groups. For positive one-point serology, the reported proportion increased from 6% in 1993, to 10% in 1994, 12% in 1995, and 27% to 29% in 1996 and 1997. The discrepancy was somewhat greater with increasing age. The increase in the reported proportions of cases with positive two-point and one-point serology was probably underestimated because the database of reported cases contained serologically confirmed cases that could not be matched with the serodiagnosis database. This proportion was highest in 1997 (Figure 3).

### Discussion

The 1996 surveillance data show that the unexpected pertussis outbreak was detected not only by increased reporting of cases (5) but also by increased hospitalizations, cases with positive serology, and deaths. Vaccine effectiveness, which had already declined in 1994 and 1995, declined further in 1996 and 1997. According to case reports and serologic data, in 1996 the increase in pertussis incidence among (mostly unvaccinated) children <1 year of age was similar to the increase in hospitalizations. However, in older, mostly vaccinated persons the increase in hospitalizations was relatively small. Contrary to reports at the time, a somewhat smaller epidemic likely occurred in 1986 and 1987 (16,17).

The surveillance data for pertussis in the Netherlands were affected by changes in availability and interpretation of serologic tests, case definitions for reporting, and case-reporting rate. However, by relying on various surveillance sources, applying criteria for one-point serology used in recent years to serologic data of 1986 and 1987, and matching our database of reported cases with our serodiagnosis database, we gained a better understanding about whether observed changes in surveillance data represented true changes in the underlying incidence of pertussis.

The trend in hospitalizations likely reflects the incidence of severe pertussis; however, this trend is probably less sensitive to changes in availability and interpretation of serologic tests, case definitions, and case-reporting rate. Thus, increasing or decreasing reports of pertussis case and data on positive serology are likely to (at least partially) reflect true changes when they are accompanied by similar trends in hospitalizations.

We obtained more insight into the effect of changes in definitions of positive serology and case definitions for reporting on serologic and reported data. The current, more restrictive, criteria for positivity of one-point serology were applied to serodiagnostic data of 1986 and 1987. It was possible to study changes in the rate of reported cases with positive one-point serology and cases with positive two-point serology in 1993 to 1997 by linking the case-reporting and serodiagnosis databases. Furthermore, stratifying case reports according to method of diagnosis led us to conclude that the decrease in estimated vaccine effectiveness and the shift in 1996 and 1997 toward older age groups in the reported cases could only partly be explained by the enhanced application of positive one-point serology. Because of great variation in case definitions and types of laboratory confirmation, comparing numbers of reported cases in different countries is meaningless. Hospitalizations, although limited to severe pertussis cases, might be more useful for such international comparisons.

Our results clearly show that pertussis has remained endemic with epidemic peaks in the Netherlands, despite high vaccination coverage. Immunity after infection, as well as after vaccination, is not lifelong. Waning vaccine-induced immunity has been suggested as an explanation for the reemergence of the disease in other countries and probably has contributed to the pertussis epidemic in the 1980s and in 1996-97 (18-20). However, the outbreak in 1986-87 may also have been associated with the Dutch vaccine's temporary reduction in potency—from 16 to 10 opacity units per dose—in 1976 to 1984. The somewhat lower vaccine-effectiveness estimates in 1986 and 1987 might be explained by greater exposure to *B. pertussis* in epidemics than in interepidemic periods (21,22).

The remarkable increase in reported cases among vaccinated patients over a wide age range, starting 2 years before the 1996 outbreak, suggests a mismatch between circulating strains and vaccine strains (5,7-9). Antigenic divergence between vaccine strains and clinical isolates was observed for two important protective antigens, pertactin and pertussis toxin (9). Furthermore, data suggest that the whole-cell vaccine protects better against strains with the pertactin vaccine type than against strains with nonvaccine types (9).

By analyzing serologic and hospitalization data apart from case-reporting data, we assessed the increase in pertussis incidence in 1996 among (mostly unvaccinated) children <1 year of age. The increase in incidence was accompanied by a similar increase in hospitalizations for pertussis in the same age group, which indicates that the virulence of *B. pertussis* for unvaccinated and unexposed persons did not change during the outbreak.

In contrast, for older, mostly vaccinated persons, the increase in hospitalizations was smaller than the increase found in other surveillance sources. While the incidence of hospital admissions was highest for infants <1 year of age, the incidence in other surveillance sources was highest in 4-year-old children. Therefore, a greater proportion of infected vaccinated persons may have had clinical symptoms because of antigenic shifts, which probably led to greater transmission of bacteria and thus a greater degree of infection in the population. This is shown by the increase of cases in unvaccinated infants.

Despite the findings of antigenic variants of pertactin and pertussis toxin in other countries, no outbreaks similar to those in the Netherlands have been observed (23-25). The vaccines in these countries may be potent enough to offset antigenic variation, or pertussis vaccines may protect less well against strains with pertactin profiles dominant in the Netherlands but less common elsewhere (23). The Dutch vaccine has been used in the National Immunization Program since 1953. No sign of an abrupt deterioration of vaccine quality, as determined for product release by the mouse protection test, has been found (5). However, the Dutch whole-cell vaccine induces low levels of antibodies against pertussis toxin and filamentous hemagglutinin and high levels of antibodies to agglutinogens and pertactin (6). This immunogenicity profile may have resulted in a greater vulnerability of the vaccinated Dutch population to antigenic changes in *B. pertussis,* especially with respect to pertactin. Since November 1997, the production process for the Dutch pertussis vaccine has been improved, resulting in a slightly higher expression of pertussis toxin. We have not yet studied the potential effect of this change.

Pertussis is also reemerging in Canada, where similar surveillance patterns may elucidate the role of the vaccine's immunogenicity profile (1,2,5). These similarities are the small proportion of infants and large proportion of patients 1 to 9 years of age affected, estimates of low vaccine effectiveness, and lower levels of antibodies against pertussis toxin after vaccination with the Canadian whole-cell vaccine (20,26). By contrast, the increase in pertussis cases in the United States is accompanied by greater proportions of affected infants and adults and more favorable vaccine-effectiveness estimates (22,27,28). The levels of pertussis toxin antibodies after vaccination were higher for an American whole-cell vaccine than for Canadian whole-cell vaccines (26).

### Conclusions

The surveillance data support, even if they do not definitively explain, the hypothetical role of antigenic changes in *B. pertussis* during the 1996 pertussis outbreak in the Netherlands. The indisputable role of whole-cell vaccine in protecting against severe pertussis is clearly shown by the sharp decrease in hospitalizations among children >12 months of age; this protection is also shown by a much smaller pertussis incidence (in the past and at present) in the Netherlands than in countries with large unvaccinated populations (29). In such countries, 60% of unvaccinated persons have clinical pertussis before the age of 10. This incidence is at least 30 times higher than that in the Netherlands, even if we assume a case-reporting rate of 25% and an incidence similar to that observed during the Dutch epidemic in 1996.

Booster vaccination will be helpful in reducing the incidence of pertussis. However, some acellular vaccines do not contain the antigenic variants of pertactin and pertussis toxin that dominate in Europe (9,23,24). Furthermore, if pertussis infections are to be postponed until adulthood as a result of boosting, the probability of transmission from adults to young, unvaccinated infants might be greater. The effects of booster vaccination on the epidemiology of pertussis must be monitored carefully, and various surveillance sources must be used to distinguish surveillance artifacts and real epidemiologic effects.

### Acknowledgments

We thank H.G.L. Boshuis and G.N.M. Aelbers for supplying data from the serodiagnostic database.

Dr. de Melker is an epidemiologist in the Department of Infectious Diseases Epidemiology, National Institute of Public Health and the Environment, the Netherlands. Her work involves epidemiologic research directed to vaccine-preventable diseases and evaluation of the National Vaccination Program.

### References

1. De Serres G, Boulianne N, Douville Fradet M, Duval B. Pertussis in Quebec: ongoing epidemic since the late 1980s. Can Commun Dis Rep 1995;21:45-8.
2. Milord F. Resurgence of pertussis in Monteregie, Quebec-1990-1994. Can Commun Dis Rep 1995;21:40-4.
3. Centers for Disease Control and Prevention. Resurgence of pertussis--United States, 1993. MMWR Morb Mortal Wkly Rep 1993;42:952-3, 959-60.
4. Andrews R, Herceg A, Roberts C. Pertussis notifications in Australia, 1991 to 1997. Commun Dis Intell 1997;21:145-8.
5. de Melker HE, Conyn-van Spaendonck MAE, Rümke HC, Wijngaarden JK van, Mooi FR, Schellekens JFP. Pertussis in the Netherlands: an outbreak despite high levels of immunization with whole cell vaccine. Emerg Infect Dis 1997;3:175-8.
6. Labadie J, Sundermann LC, Rümke HC, and the DTP-IPV-Hib vaccine study group. Multi-center study on the simultaneous administration of DTP-IPV and Hib PRP-T vaccines. Part 1. Immunogenicity. Bilthoven, The Netherlands: National Institute of Public Health and the Environment; 1996. Report No. 124001003.
7. van der Zee A, Vernooij S, Peeters M, van Embden J, Mooi FR. Dynamics of the population structure of *Bordetella pertussis* as measured by IS1002-associated RFLP: comparison of pre- and post-vaccination strains and global distribution. Microbiology 1996;142:3479-85.
8. Mooi FR, van Oirschot H, Peeters J, Willems RJL. Antigenic variation of the acellular vaccine component pertactin in the Dutch *B. pertussis* population. Bilthoven, The Netherlands: National Institute of Public Health and the Environment; 1995. Annual Report p. 74-5.
9. Mooi FR, van Oirschot H, Heuvelman K, van der Heide HGJ, Gaastra W, Willems RJL. Polymorphism in the *Bordetella pertussis* virulence factors P.69/pertactin and pertussis toxin in the Netherlands: temporal trends and evidence of vaccine-driven evolution. Infect Immun 1998;66:670-5.
10. Nagel J, Poot-Scholtens EJ. Serum IgA antibody to *Bordetella pertussis* as an indicator of infection. J Med Microbiol 1983;16:417-26.
11. Nagel J, de Graaf S, Schijf-Evers D. Improved serodiagnosis of whooping cough caused by *Bordetella pertussis* by determination of IgG anti-LPF antibody levels. Dev Biol Stand 1985;61:325-30.
12. de Melker HE, Boshuis HGL, Mommers MPTM, Sundermann LC, Labadie J, Rümke HC, et al. Serodiagnostiek van kinkhoest: interpretatie van hoge IgG/IgA concentraties in acute fase serum. (Her)evaluatie van eenpuntsserologie. Bilthoven, The Netherlands: National Institute of Public Health and the Environment; 1996. Report No. 128507003.
13. van der Zee A, Agterberg C, Peeters M, Mooi F, Schellekens J. A clinical validation of *Bordetella pertussis* and *Bordetella parapertussis* polymerase chain reaction: comparison with culture and serology using samples from patients with suspected whooping cough from a highly immunized population. J Infect Dis 1996;174:89-96.
14. de Melker HE, Versteegh FGA, Conyn-van Spaendonck MAE, Elvers LH, Berbers GAM, van der Zee A, et al. Specificity and sensitivity of high levels of IgG antibodies against pertussis toxin in a single serum for diagnosis of infection with *Bordetella pertussis.* J Clin Microbiol 2000;38:800-6.
15. Farrington CP. Estimation of vaccine effectiveness using the screening method. Int J Epidemiol 1993;22:742-6.
16. van de Water HPA. Kinkhoestaangiften in Nederland in de periode 1976-1986 en de noodzaak van een uniforme ziektedefinitie. Ned Tijdschr Geneeskd 1988;132:821-8.
17. van de Water HPA, van de Laar MJW, Leentvaar-Kuijpers A. Wanneer is het kinkhoest? Verband tussen laboratoriumdiagnostische bevindingen en klachten. Ned Tijdschr Geneeskd 1988;132:828-33.
18. Bass JW, Stephenson SR. The return of pertussis. Pediatr Infect Dis J 1987;6:141-4.
19. Bass JW, Wittler RR. Return of epidemic pertussis in the United States. Pediatr Infect Dis J 1994;13:343-5.
20. Bentsi-Enchill AD, Halperin SA, Scott J, MacIsaac K, Duclos P. Estimates of effectiveness of a whole-cell pertussis vaccine from an outbreak in an immunized population. Vaccine 1997;15:301-6.
21. Ramsay MEB, Farrington CP, Miller E. Age-specific efficacy of pertussis vaccine during epidemic and non-epidemic periods. Epidemiol Infect 1993;111:41-8.
22. Guris D, Strebel PM, Tachdjian R, Bardenheier B, Wharton M, Hadler SC. Effectiveness of the pertussis vaccination program as determined by the use of the screening method: United States, 1992-1994. J Infect Dis 1997;176:456-63.
23. Mooi FR, He Q, van Oirschot H, Mertsola J. Variation in *Bordetella pertussis* virulence factors pertussis toxin and pertactin in vaccine strains and clinical isolates in Finland. Infect Immun 1999;67:3133-4.
24. Mastriantonio P, Spigaglia P, van Oirschot H, van der Heide HGJ, Heuvelman K, Stefanelli P, et al. Antigenic variants in *Bordetella pertussis* strains isolated from vaccinated and unvaccinated children. Microbiology 1999;145:2069-75.
25. Guiso N. Can new vaccines meet the challenge of novel pertussis strains? Abstract book of the 17th Annual Meeting of the European Society for Paediatric Infectious Diseases. Greece; 1999 May 19-21; p. 10.

26. Baker JD, Halperin SA, Edwards K, Miller B, Decker M, Stephens D. Antibody response to *Bordetella pertussis* antigens after immunization with American and Canadian whole-cell vaccines. J Pediatr 1992;121:523-7.
27. Centers for Disease Control and Prevention. Pertussis—United States, January 1992-June 1995. MMWR Morb Mortal Wkly Rep 1995;44:526-9.
28. Guris D, Strebel PM, Bardenheier B, Brennan M, Tachdjian R, Finch E, et al. Changing epidemiology of pertussis in the United States: increasing reported incidence among adolescents and adults, 1990-1996. Clin Infect Dis 1999;28:1230-7.
29. Isacson J, Trollfors B, Taranger J, Zackrisson G, Lagergard T. How common is whooping cough in a nonvaccinating country? Pediatr Infect Dis J 1993;12:284-8.
